# Proteomic profiling identifies prognostic signature for Krukenberg tumor of gastrointestinal origin

**DOI:** 10.1016/j.isci.2026.114682

**Published:** 2026-01-13

**Authors:** Xiaoling Wang, Xiao Yi, Zhangzhi Xue, Wei Liu, Xiaodong Teng, Han Zhang, Tiannan Guo, Yi Zhu, Bo Wang

**Affiliations:** 1 Department of Pathology, The First Affiliated Hospital, Zhejiang University School of Medicine, Hangzhou, Zhejiang, China; 2 School of Medicine, School of Life Sciences, Westlake University, Hangzhou, Zhejiang, China; 3 Westlake Center for Intelligent Proteomics, Westlake Laboratory of Life Sciences and Biomedicine, Hangzhou, Zhejiang, China; 4 Westlake Omics (Hangzhou) Biotechnology Co., Ltd., Hangzhou 310024, China

**Keywords:** Cancer, Proteomics

## Abstract

Krukenberg tumor (KT) primarily originates from the stomach and colorectum, but reliable biomarkers for distinguishing KT from other tumors at the same sites and predicting ovarian metastasis remain lacking. Using pressure cycling technology (PCT) and data-independent acquisition (DIA) mass spectrometry, we analyzed 263 formalin-fixed paraffin-embedded (FFPE) samples, identifying 10,837 proteins. The results revealed distinct proteomic signatures from the primary gastrointestinal lesions of KT. Comparative analyses identified group-specific pathways, particularly mesenchymal-epithelial transition (MET) signaling pathways and extracellular matrix (ECM) pathways, that were enriched in the primary lesions of KT. We developed protein-based classifiers with promising diagnostic value in distinguishing the primary gastrointestinal lesions of KT from those without ovarian metastases. We depicted distinct proteomic signatures in the primary gastrointestinal lesions of KT and identified potential biomarkers for prediction and early intervention of gastrointestinal cancer patients at risk of ovarian metastases.

## Highlights

• Identification of proteomic signatures provides valuable characterization of KT
• Protein-based classifiers can aid in accurate identification and classification of KT
• ECM pathways identify therapeutic targets for GI cancer with ovarian metastases

## Graphical abstract

**Figure:**
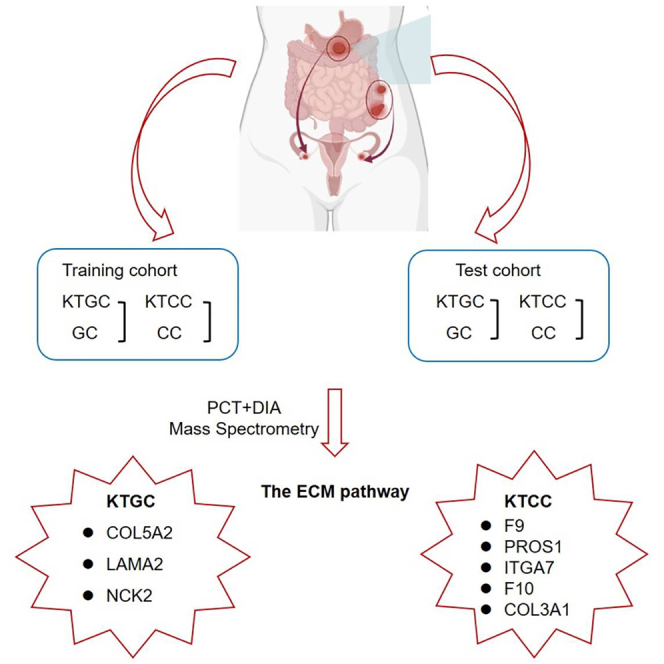

## Introduction

Krukenberg tumor (KT) is an uncommon metastatic tumor of the ovary containing a signet ring cell component, accounting for 1%–2% of all ovarian tumors.^1^ This distinctive tumor was named after Friedrich Krukenberg (1871–1946), a German gynecologist and pathologist. According to reports, the majority of KT cases originate in the stomach (76%), followed by the colorectal tract (11%).^2^ It remains unclear how tumors disseminate and inflict harm upon their host organisms. However, the organ distribution of metastases from a primary tumor is not random. Based on autopsy analyses of secondary cancers in a cohort of breast cancer patients, Paget proposed the “seed and soil” hypothesis in 1889, which elaborated that particular tumor types had a predisposition to specific secondary sites.^3^

To date, there are no uniform guidelines for the treatment of KT worldwide.^4^ The main treatments for KT include prophylactic oophorectomy, surgical resection, cytoreductive surgery combined with hyperthermic intraperitoneal chemotherapy, and systematic therapy.^5^ However, the prognosis of patients with KT is still dismal, as it is always diagnosed at an advanced stage.^1^ Furthermore, the prognosis of patients with KT has been reported to vary depending on its origins.^6^ Patients with KT of genital tract origin have a better prognosis than those with extragenital tract origin.^7^ Hence, understanding the mechanisms underlying KT metastasis from the primary site and its specific origin may assist in devising targeted treatment strategies.

With the development of modern molecular biology technologies such as next-generation sequencing (NGS), potential molecular mechanisms of the occurrence of KT have been studied. Primary tumor biopsies or resected tumor tissues can be used for pathological examination as well as exploration in terms of molecular biological features, thus predicting ovarian metastasis by assessing the expression of tumor-associated genes in primary gastrointestinal cancer is promising.^8^ For instance, it has been reported that mutations in genes such as KRAS, SMAD4, NTRK1, BRAF, and RAS, as well as overexpression of HER2, may contribute to the metastasis of colorectal cancer to the ovaries.^9^^,^^10^^,^^11^^,^^12^ Distinguishing the gastrointestinal tumors with and those without ovarian metastases is crucial for the early detection of KT in the clinic. However, due to the great pathomorphological and genomic similarities shared by the two tumor entities, diagnostic biomarkers have been lacking for KT of gastrointestinal origin so far.

Different from gene expression, protein expression indicates the real functional state of an organism. Proteomics measures the whole proteome of a cell, tissue, or organism and has been widely used in the field of precision medicine, particularly cancer biomarkers for diagnosis, prognosis, drug targets, vaccine development, and exploring pathogenicity mechanisms.^13^^,^^14^^,^^15^^,^^16^ However, the proteomic events of gastrointestinal cancer transformation have not been fully described yet. Besides, there are few proteomics studies investigating protein expression patterns of primary cancers with or without ovarian metastasis. Only a limited number of studies have reported the characteristics of cancer metastasis specifically at the levels of immune-related markers, gene mutations, and proteomics. These findings contribute to a deeper understanding of the mechanisms underlying cancer metastasis while offering novel insights and potential biomarkers for the diagnosis, prognostic evaluation, and therapeutic management of associated cancers. Concurrently, they underscore the critical value of multi-dimensional research approaches in advancing the field of cancer metastasis research.^17^^,^^18^^,^^19^

In this study, we aimed to identify differential diagnostic biomarkers for KT of gastrointestinal origin. In the present work, we collected samples from gastrointestinal cancer patients with and without ovarian metastasis, and systematically compared the proteomes of the two cancer types, to explore metastasis-related potential protein biomarkers that assist early diagnosis of KT. We identified distinct proteomic signatures in the primary gastrointestinal lesions of KT. Additionally, we developed protein-based classifiers that showed promising potential in distinguishing the primary gastrointestinal lesions of KT from those without ovarian metastases. Particularly, higher expression of COL5A2 and LAMA2 was significantly associated with poorer overall survival (OS) in gastric cancer, while higher expression of ITGA7 was significantly associated with poorer OS in colorectal adenocarcinoma. Our study provides valuable insights into the molecular mechanisms of KT formation and identifies potential biomarkers for early intervention in gastrointestinal cancer patients at risk of ovarian metastases.

## Results

### Study design

**Figure 1:**
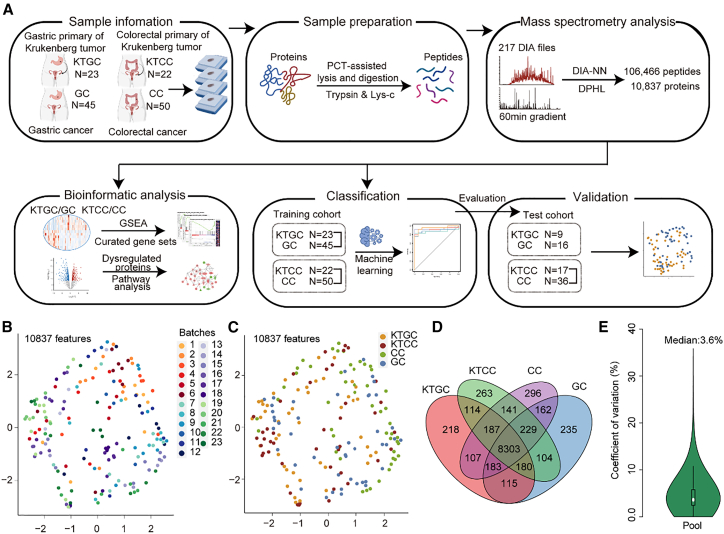
Overview of the study (A) Study design and analysis pipeline. UMAP analysis was performed on 10,837 proteins and colored by 23 batches (B) or four tumor types (C). (D) Venn diagram showing the overlap among four protein sets. (E) The violin plot shows the distribution of CV values for protein intensity in the pooled samples.

In this study, we first collected primary tumor with KT of gastric origin (KTGC) from 23 patients and primary tumor with KT of colorectal origin (KTCC) from 22 patients, respectively. As a comparison, we also included primary tumor tissue specimens from 45 patients with gastric cancers (GC) and 50 patients with colorectal cancers (CC) in the training cohort as the control groups. No evidence of ovarian metastasis was found in these control patients during the same monitoring period (Figure 1A). Representative hematoxylin and eosin (H&E) images of KT and corresponding primary cancers are presented in Figure S1. Detailed clinical characteristics of the patients are available in Table S3.

Altogether, we obtained a total of 185 formalin-fixed paraffin-embedded (FFPE) tumor tissue samples (including 45 biological replicates) from 140 patients and performed PCT-assisted protein lysis and peptides extraction, followed by data-independent acquisition (DIA)-based mass spectrometry data acquisition. As detailed in Table S4, using DIA-NN,^20^ we quantified 10,837 unique proteins (per sample range: 2,934–6,259; median = 5,191) derived from 106,466 prototypic peptides (per sample range: 15,217–39,096; median = 28,761.5). Based on proteomics data, we conducted an in-depth analysis to explore the differentially expressed proteins and pathways of KTGC vs. GC, and KTCC vs. CC, respectively. Besides, we also constructed machine learning-based classification models to distinguish KTGC from GC, and KTCC from CC, respectively. A second independent test cohort, including 9 KTGC patients and 16 GC patients, 17 KTCC patients and 36 CC patients, was used to evaluate the robustness of the models.

### Clinicopathological features of patients

Within the training cohort, all KTGC patients were aged below 60 years, with an average age of 42.0 years at primary cancer diagnosis (ranging from 24 to 57 years). A majority, 20 (87.0%) were premenopausal, and 3 (13.0%) were postmenopausal. Primary cancer and KT detection occurred simultaneously in 12 (52.2%) patients. In the remaining 11 (47.8%) cases, the interval from primary cancer detection to KT identification varied from 8 to 102 months, averaging 30.3 months. Fifteen (65.2%) patients experienced bilateral ovarian involvement. Seventeen (73.9%) primary GCs were presented with signet-ring cells. All primary cancers were classified as poorly differentiated. Lymph node metastasis was confirmed in 21 (91.3%) cases (Tables S1 and S3).

For the KTCC patients, 17 out of 22 were under 60, with an average age at primary cancer diagnosis of 45.9 years, ranging from 28 to 70 years. Of these, 15 (68.2%) patients were premenopausal, and 7 (31.8%) were postmenopausal. Primary cancer and KT detection were synchronous in 12 (54.5%) patients. For the remaining 10 (45.5%) cases, the interval between primary cancer detection and KT ranged from 8 to 32 months, averaging 12.5 months. Fourteen (63.6%) patients experienced bilateral ovarian involvement. Signet-ring cells were present in 2 (9.1%) primary cancers. Thirteen (59.1%) primary cancers were poorly differentiated. Lymph node metastasis was confirmed in 17 (77.3%) cases (Tables S2 and S3).

In the test cohort, all nine KTGC patients were aged below 60 years, with an average age at primary cancer diagnosis of 48.4 years, ranging from 43 to 56 years. Six (66.7%) patients were premenopausal, and eight (88.9%) patients exhibited metachronous cancers. The interval between primary cancer detection and KT identification ranged from 5 to 58 months, averaging 20.1 months. Bilateral ovarian involvement was observed in eight (88.9%) patients. Signet-ring cells were absent in most cases (*n* = 6), and all the primary cancers were poorly differentiated and showed positive lymph node metastasis (Table S3).

Among the patients in the test group of KTCC, the average age at primary cancer diagnosis was 54.4 years (range 36–73 years), with ten patients under 60. Seven (41.2%) were premenopausal, and 10 (58.8%) were postmenopausal. Ovarian metastasis was confirmed at the initial primary cancer diagnosis in eight (47.1%) patients. For the other nine (52.9%) cases, the interval from primary cancer detection to KT ranged from 5 to 46 months, averaging 15.2 months. Five (29.4%) patients showed bilateral ovarian metastasis at presentation. Signet-ring cells were present in only one (5.9%) patient, while moderate differentiation was more common (*n* = 13). Lymph node metastasis was detected in 14 (82.4%) patients (Table S3).

Table S3 depicts more detailed information of the other 45 cases of GC and 50 cases of CC in the training cohort and the 16 cases of GC and 36 cases of CC in the test cohort.

### Quality control analysis of the proteome data

To minimize batch effects, we divided the 185 samples into 23 batches randomly. According to manifold approximation and projection (UMAP) analysis based on 10,837 characterized proteins, batch effects are minimal (Figure 1B). However, different tissues cannot be distinguished in the UMAP (Figure 1C). As shown in Figure 1D, these four groups share 8,303 overlapping proteins, (which contributes to 76.6%) resulting in the unclear separation by UMAP. Additionally, the small sample size further limits the ability of UMAP to distinguish these groups. To assess the reproducibility of our proteomics data, we randomly selected nine samples for repeated DIA injections as technical replicates. Besides, a pooled sample, named as “pool,” was assigned to each batch for evaluating the DIA-MS analysis. The Pearson correlation coefficients of 23 pools and the nine technical replicated pairs were all above 0.9, confirming the high degree of technical reproducibility (Figures S2A and S2B). The median coefficient of variation (CV) of protein intensity across 23 pools was 3.6%, further affirming the consistency and reliability of our data (Figure 1E).

### Mesenchymal-epithelial transition signaling pathway and extracellular matrix organization may contribute to KTGC

To investigate the mechanisms underlying the formation of gastric-origin KT, we conducted a comparative analysis of proteomic profiles between primary gastric tumor tissues with and without ovarian metastases, KTGC versus GC.

**Figure 2:**
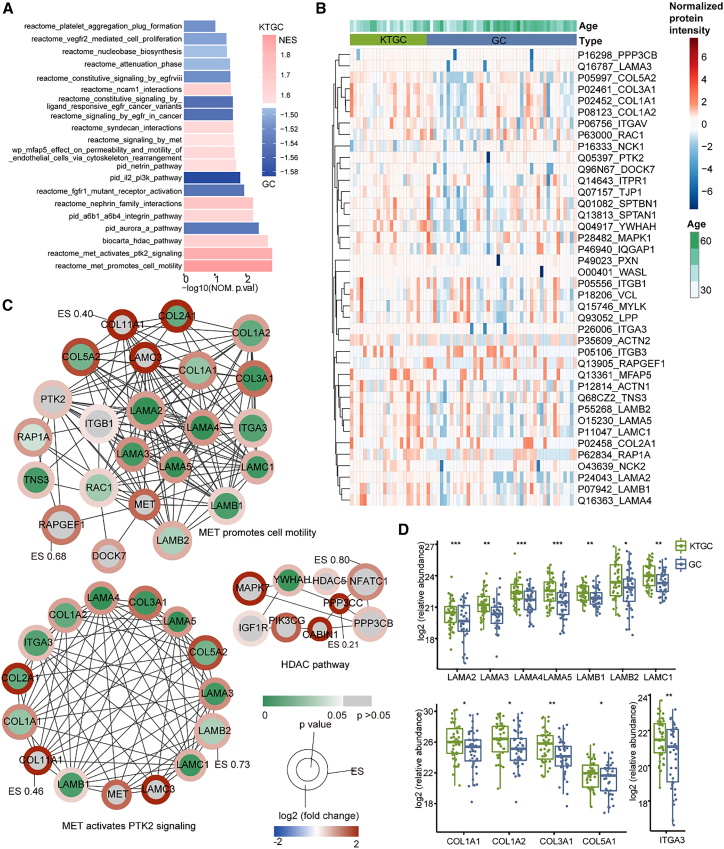
Differential analysis of KTGC and GC proteome (A) GSEA enriched processes specific to KTGC and GC, with NES and nominal *p* values shown. The nominal *p* value (NOM.p.val) of each pathway represents the statistical significance of the enrichment score. (B) The heatmap displays proteins involved in the top five enriched pathways. The proteins with over 70% missing ratios were removed. (C) Network representations of proteins from the top three enrichment pathways and visualized by Cytoscape. Protein-protein interactions were annotated by the String database. (D) Boxplots display the protein expression in KTGC and GC. The *p* value was calculated by a two-sided unpaired *t* test using log2 relative protein abundance between groups. *p* value: ∗*p* < 0.05; ∗∗*p* < 0.01; ∗∗∗*p* < 0.001.

We first performed pathway-level gene set enrichment analysis (GSEA) to identify pathways that were specific to each group. The top 10 pathways, ranked by the absolute value of the normalized enrichment score (NES), enriched in KTGC are mainly associated with the MET signaling pathway and extracellular matrix (ECM) organization (Figure 2A). The most enriched pathways of KTGC were associated with MET signaling, i.e., MET promotes cell motility, MET activates PTK2 signaling, and skeletal myogenesis (HDAC pathway) (Figures 2A–2C). These pathways have been previously described to be associated with tumor metastasis.^21^^,^^22^^,^^23^^,^^24^ Among the proteins involved in these three pathways, we identified significant upregulation of three protein families in KTGC. These protein families include laminins (LAMA2, LAMA3, LAMA4, LAMA5, LAMB1, LAMB2, and LAMC1), collagens (COL1A1, COL1A2, COL3A1, and COL5A1), and integrin (ITGA3) (Figure 2D).

Differentially expressed analysis revealed a total of 154 proteins that exhibited significant protein abundance difference between these KTGC and GC tumors (adjusted *p* value <0.05 adjust by BH method and |log_2_ (fold-change) | >0.5). Among these proteins, 133 were upregulated and 21 were downregulated in the KTGC tumors (Figure 4A and Table S5). The upregulated proteins are primarily associated with ECM organization, laminin interactions, degradation of the ECM, hemostasis, and retrograde endocannabinoid signaling (Figures S3B and S3D), which was consistent with the findings from the GSEA analysis. On the other hand, the downregulated proteins are involved in pathways such as cellular responses to stress, negative regulation of supramolecular fiber organization, metabolism of RNA, cytokine signaling in the immune system, and regulation of DNA metabolic process (Figures S3C and S3E).

Overall, our findings suggest that the MET signaling pathway and ECM organization might play crucial roles in the formation of gastric-origin KT. The upregulation of specific proteins within these pathways, such as laminins, collagens, and integrin, may contribute to the metastatic behavior of these tumors.

### Similarly dysregulated pathways in KTCC and KTGC

**Figure 3:**
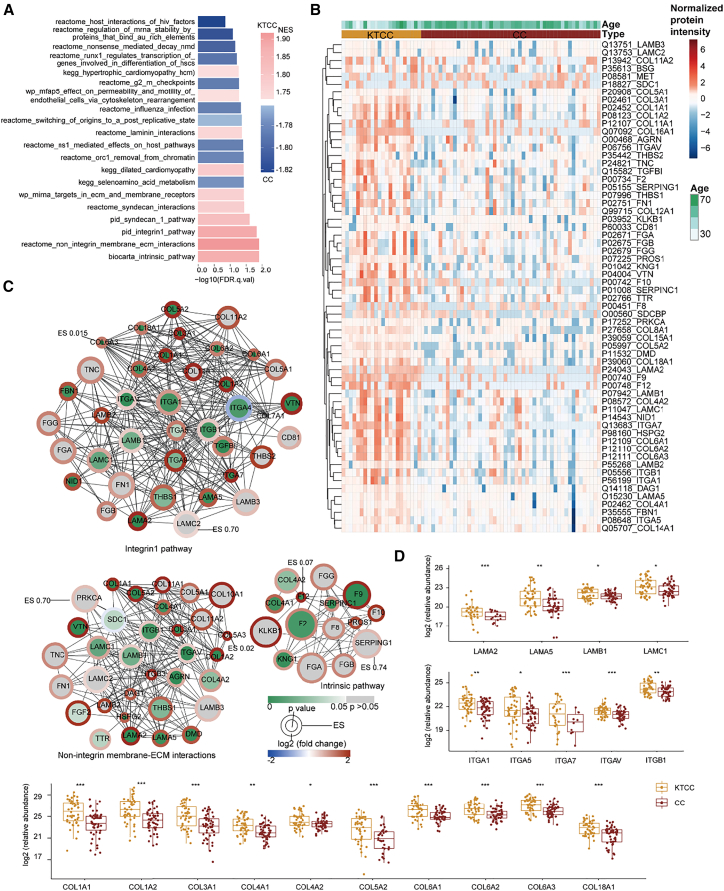
Differential analysis of KTCC and CC proteome (A) GSEA enriched processes specific to KTCC and CC, with NES and nominal *p* values shown. The nominal *p* value (NOM.p.val) of each pathway represents the statistical significance of the enrichment score. (B) The heatmap shows proteins are involved in the top five enriched pathways. The proteins with over 70% missing ratios were removed. (C) Network representations of proteins from the top three enrichment pathways and visualized by Cytoscape. Protein-protein interactions were annotated by the String database. (D) Boxplots display the protein expression in KTCC and CC. The *p* value was calculated by a two-sided unpaired *t* test using log2 relative protein abundance between groups. *p* value: ∗*p* < 0.05; ∗∗*p* < 0.01; ∗∗∗*p* < 0.001.

Similarly, pathway-level GSEA was performed to compare the KTCC and CC tumors (Figure 3A). Our findings revealed that the top 10 pathways enriched in KTCC are primarily associated with ECM organization. Additionally, the most significantly enriched pathways of KTCC are the intrinsic prothrombin activation pathway, non-integrin membrane-ECM interactions, and beta1 integrin cell surface interactions (Figures 3A–3C). Among these three pathways, we also found three family proteins, i.e., integrins (ITAG1, ITGA5, ITGA7, ITGAV, and ITGB1), collagens (COL1A1, COL1A2, COL3A1, COL4A1, COL4A2, COL5A2, COL6A1, COL6A2, COL6A3, and COL18A1), and laminins (LAMA2, LAMA5, LAMB1, and LAMC1). They are upregulated in KTCC, which is consistent with the observations in the KTGC cases (Figures 2D and 3D).

**Figure 4:**
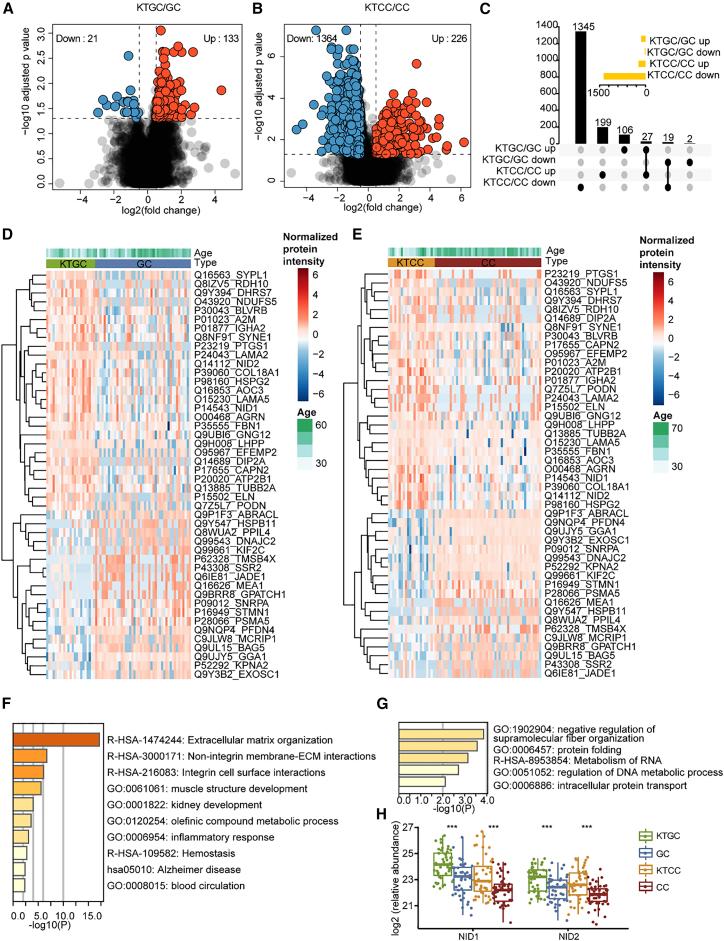
Comparison of differentially expressed proteins in KTGC/GC and KTCC/CC groups The volcano plot depicts significantly regulated proteins in KTGC/GC (A) or KTCC/CC (B). Proteins with adjusted *p* value (<0.05) adjust by the BH method and |log2 (fold-change) | >0.5 are colored blue or red. (C) Visualization of intersections of significantly regulated proteins from A and B. Heatmap displays the overlapped regulated protein expression in KTGC/GC and KTCC/CC (D–E). The bar plot showed the enriched pathways by Metascape using overlapped upregulated (F) or downregulated (G) proteins in KTGC and KTCC. (H) Boxplots display the protein expression in KTGC, GC, KTCC, and CC. *p* value: ∗*p* < 0.05; ∗∗*p* < 0.01; ∗∗∗*p* < 0.001.

The differential analysis of protein expressions between KTCC and CC identified 1,590 significantly dysregulated proteins (Figure 4B and Table S5). Of these, 226 proteins are upregulated in KTCC, and the main pathways involved are NABA CORE MATRISOME, ECM organization, complement and coagulation cascades, hemostasis, and NABA BASEMENT MEMBRANES (Figures S4B and S4D). The other 1,364 proteins are downregulated in KTCC, and the main pathways involved include the metabolism of RNA, mRNA metabolic process, translation, and rRNA processing (Figures S4C and S4E). Notably, the enriched pathways from the differentially expressed proteins in the KTCC tumors exhibited a high degree of consistency with those observed in the KTGC tumors. Specifically, the upregulated proteins are predominantly associated with ECM pathways (Figure S5), while the downregulated proteins are involved in the metabolism of RNA pathway. This observation implies that gastric and CCs developing ovarian metastases exhibit similar dysregulated signaling pathways.

### Comparison of differentially expressed proteins in the KTGC/GC and KTCC/CC tumors

To further investigate the formation mechanism of KT of different origins, we conducted a comparative analysis of the two regulated proteomes, between KTGC/GC and KTCC/CC. We identified 27 upregulated proteins and 19 downregulated proteins shared between the two groups (Figures 4A–4E). The 27 upregulated proteins were primarily related to the ECM pathway, (Figure 4F). The 17 downregulated proteins were involved in the negative regulation of supramolecular fiber organization and RNA transcriptional regulation-related pathways (Figure 4G).

Notably, the nidogen family proteins (NID1, NID2) are upregulated in both KTGC and KTCC tumors (Figure 4H). Similar to the three other protein families (integrins, collagens, and laminins), nidogens are also associated with ECM. Studies have demonstrated that the upregulation of NID1 and NID2 is associated with metastasis and poor prognosis in various cancers, including GC, ovarian cancer, salivary gland adenoid cystic carcinoma, and hepatocellular carcinoma.^25^^,^^26^^,^^27^^,^^28^^,^^29^ Several studies have identified NID1 as a novel regulator affecting epithelial-mesenchymal transition (EMT), a cell biological process that allows epithelial cells to acquire mesenchymal cell characteristics, thereby enhancing tumor cell migration and invasion.^27^^,^^28^^,^^30^ NID1 promotes EMT in tumor cells by activating the ERK/MAPK or PI3K/AKT signaling pathways. ECM reorganization and altered cellular responses to ECM are critical for EMT activation and evolution.^31^ The ECM pathway has been implicated in tumor migration, invasion, and induction of angiogenesis and is considered a therapeutic target for cancer.^32^

Therefore, ECM and EMT activation may play a critical role in the formation of KT by facilitating cancer cells from the gastrointestinal tract to invade and metastasize to the ovaries. Further research is needed to fully understand the underlying mechanisms of EMT in KT formation and progression.

### Machine learning and the development of predictive protein classifiers for KT

Based on the characteristic protein profiles of KTGC/GC and KTCC/CC, we sought to find potential predictive biomarkers for the formation of KT.

**Figure 5:**
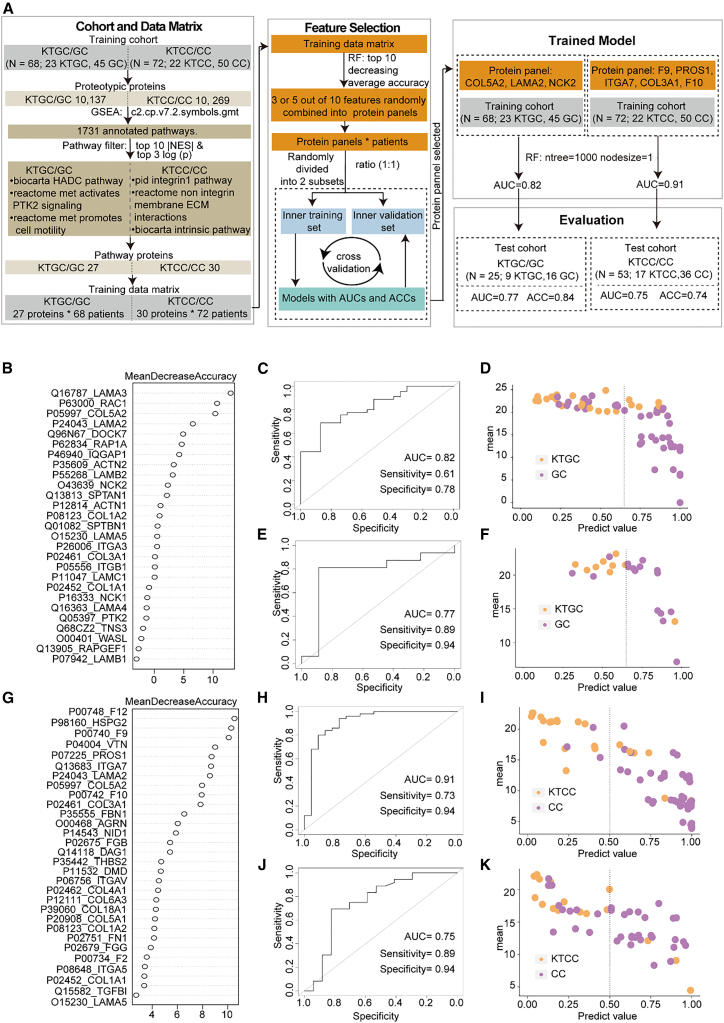
Separation of KTGC, KTCC and GC, CC patients via machine learning of proteomics features (A) Workflow for generating models to distinguish KTGC and GC, KTCC and CC. (B) The top 27 proteins prioritized by random forest analysis are ranked by mean decrease in accuracy for separating KTGC and GC. (G) The top 30 proteins prioritized by random forest analysis are ranked by mean decrease in accuracy for separating KTCC and CC. Receiver operating characteristic (ROC) of the random forest model in the training cohort of KTGC/GC (C) and KTCC/CC (H). Performance of the model in the training cohort of KTGC/GC (D) and KTCC/CC (I). ROC of the random forest model in the test cohort of KTGC/GC (E) and KTCC/CC (J). Performance of the model in the test cohort of KTGC/GC (F) and KTCC/CC (K).

Regarding the cases of KTGC, as demonstrated above, 23 KTGC patients and 45 GC patients were used as the training cohort. We employed random forest analysis for protein feature selection (Figure 5A). First, we ranked 27 proteins from the three pathways in Figure 2A according to the decreasing average accuracy and selected the top 10 proteins as candidate proteins for building models (Figure 5B). Then, we constructed a three-protein classifier (COL5A2, LAMA2, and NCK2) to distinguish patients of KTGC from patients of GC. The LAMA2 protein demonstrated statistically significant differential expression (*p* < 0.05) in the comparative analysis between the KTGC and GC groups. The model achieved an area under the curve (AUC) of 0.82 in the training cohort (Figures 5C and 5D). Thereafter, a test cohort comprising 9 KTGCs and 16 GCs was collected to verify the applicability of this model. The model achieved an AUC value of 0.77 in the test cohort, with 21 out of 25 patients correctly classified, resulting in an accuracy rate of 0.84 (Figures 5E and 5F).

Regarding the case of KTCC, we employed a training cohort of 22 KTCC patients and 50 CC patients, along with a test cohort of 17 KTCC patients and 36 CC patients. Random forest analysis was performed for protein feature selection, and the top 30 proteins from the three pathways in Figure 3A were selected as candidate proteins based on decreasing average accuracy. Then, a five-protein classifier (F9, PROS1, ITGA7, F10, and COL3A1) was built to distinguish KTCC from CC (Figure 5G). Three of the five biomarkers (F9, ITGA7, and COL3A1) were also significantly differentially expressed (*p* < 0.05) when comparing the KTCC and CC cohorts. The classifier achieved AUC values of 0.91 in the training cohort and 0.75 in the test cohort (Figures 5H–5K). A total of 39 out of the 53 (0.74) samples in the test cohort were correctly classified. These results suggest that the protein-based model achieved well diagnostic performance in the new cohort.

Overall, our results suggest that models based on differentially expressed pathways and proteins can effectively distinguish KTGC from GC and KTCC from CC, providing promising diagnostic performance in new cohorts. These findings contribute to the identification of potential biomarkers for KT formation and may have implications for improved patient stratification and personalized treatment strategies.

## Discussion

Proteomic profiling has gained traction in the identification of risk features correlated with the progression of primary malignancies and in predicting the propensity for metastatic cancer development.^8^ In this study, we aimed to distinguish KT formation from gastric and colorectal origins and identify potential biomarkers for these metastatic tumors. Utilizing a PCT-DIA-MS proteome profiling workflow, we analyzed a total of 263 FFPE specimens derived from a cohort of 140 KT patients, representing an unprecedented scale in our existing body of knowledge. We evaluated the proteomic features and characteristics of primary lesions of KT in comparison to primary tumors at the same anatomic site but without ovarian metastases.

Through the implementation of comparative proteomic analyses and pathway-level GSEA, we identified group-specific pathways enriched in KTGC and KTCC, primarily associated with MET signaling pathways and ECM pathways. Intriguingly, we found that laminins, collagens, and integrins, which are known to be components of the ECM, were upregulated in both KTGC and KTCC. Dysregulation of these ECM proteins may promote the invasion of cancer cells into the ovaries, leading to KT formation. Encouragingly, some clinical trials are using enzymes, antibodies, or small molecules to inhibit the expression and function of proteins in the ECM components, to prevent the formation of pre-metastatic cancer microenvironments.^33^^,^^34^^,^^35^^,^^36^^,^^37^^,^^38^ Moreover, our study suggests that KTGC and KTCC exhibited similar proteomic characteristics, with the same pathways enriched in upregulated proteins related to the ECM pathway and downregulated proteins related to the metabolism of RNA pathway. These findings suggest that GC and colorectal adenocarcinoma that develop ovarian metastases may share common molecular mechanisms in the formation of KT.

Furthermore, we constructed a three-protein classifier and a five-protein classifier to distinguish KTGC and KTCC from those with gastric and CCs without ovarian metastases, respectively. These classifiers exhibited promising diagnostic performance in both the training and test cohorts, indicating their potential utility in clinical practice.

The three proteins in the predictive biomarker classifier for KTGC are COL5A2, LAMA2, and NCK2. They were all upregulated in KTGC and were involved in the ECM pathway, implying the metastatic progression of various cancers as reported. Specifically, high expression of COL5A2 has been demonstrated to promote the proliferation and metastasis of several cancers, including prostate cancer, CC, GC, breast cancer, and osteosarcoma, leading to poor prognosis, while knockdown of COL5A2 inhibits cancer progression and metastasis.^39^^,^^40^^,^^41^^,^^42^^,^^43^ Studies have also shown that upregulation of LAMA2 contributes to metastasis in clear cell renal cell carcinoma, while knockdown of LAMA2 reverses Mex3a knockdown-induced metastasis in lung adenocarcinoma.^44^^,^^45^ NCK2 has been reported to be expressed at higher levels in metastatic than non-metastatic cell lines of various cancers, such as melanoma skin cancer, colon cancer, and breast cancer.^46^^,^^47^ NCK2 can induce podosome-mediated ECM degradation in endothelial cells and promote cancer cell invasion and metastasis, while silencing of NCK2 prevents this process.^48^

The five proteins in the classifier for KTCC are F9, PROS1, ITGA7, F10, and COL3A1. They were all upregulated in the KTCC group. ITGA7 and COL3A1, like the previously mentioned ECM pathway-related model proteins, have been reported to be associated with tumor metastasis. Studies have shown that high expression of ITGA7 is associated with various aspects of tumor progression, including deteriorative tumor features, lymph node metastasis, and worse OS, in breast cancer, cervical cancer, pancreatic carcinoma, and hepatocellular carcinoma, knockdown of ITGA7 can inhibit cell proliferation and invasion while increasing apoptosis in breast cancer.^49^^,^^50^^,^^51^^,^^52^ High expression of COL3A1 is associated with distant metastasis and OS in Ewing’s sarcoma, and it is also associated with brain metastasis in breast cancer.^53^^,^^54^ Targeting COL3A1 inhibits the growth and metastasis of renal cell carcinoma and infiltration of tumor-associated macrophages.^55^ The GSEA pathway analysis results indicate that F9, F10, and PROS1 are enriched in the intrinsic pathway, suggesting that these proteins may shape the tumor microenvironment through the coagulation cascade, ultimately leading to tumor metastasis.^56^^,^^57^ Both F9 and F10 are vitamin K-dependent coagulation factors, targeted agents that specifically aim to inhibit coagulation factors are currently undergoing clinical trials as a potential treatment for metastatic solid tumors.^58^^,^^59^^,^^60^ The identified proteins in both classifiers provide insights into the mechanisms underlying KT formation and progression. They highlight the role of the ECM, tumor microenvironment, and coagulation cascade in promoting metastasis. Targeting these proteins and pathways could potentially be explored for developing therapeutic strategies to inhibit tumor metastasis in KT.

**Figure 6:**
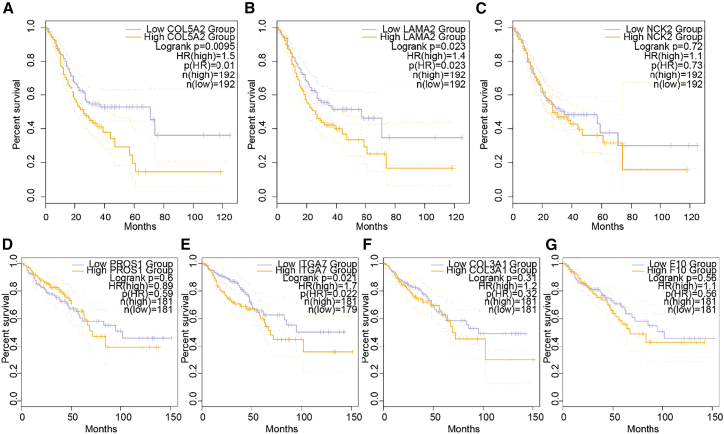
Expression and prognosis of the model genes in patients with GC and colorectal adenocarcinoma The relationship between the expression of COL5A2 (A), LAMA2 (B), and NCK2 (C) and OS in patients with GC. The relationship between the expression of PROS1 (D), ITGA7 (E), COL3A1 (F), and F10 (G) and OS in patients with colorectal adenocarcinoma.

Upon the identification of the crucial role of these proteins in discriminating against gastrointestinal cancer with or without ovarian metastasis, we further explored their prognostic significance in primary gastrointestinal cancer. We conducted a prognostic analysis for the three-protein classifier (COL5A2, LAMA2, and NCK2) in GC using GEPIA2 databases (Figures 6A–6C).^61^ Our findings revealed that higher expression levels of COL5A2 and LAMA2 were significantly associated with poorer OS in GC (*p* < 0.05). In addition, increased expression of NCK2 was also observed to be associated with poor OS in GC, although it did not reach statistical significance in the GEPIA2 dataset.

We also investigated the prognostic significance of the five-protein classifier (F9, PROS1, ITGA7, F10, and COL3A1) in colorectal adenocarcinoma using GEPIA2 (Figures 6D–6G). Our analysis showed that higher expression levels of ITGA7 were significantly associated with poorer OS in colorectal adenocarcinoma (*p* < 0.05). Except for F9, which was not found in GEPIA2 database, increased expression of PROS1, F10, and COL3A1 was also observed to be associated with poor OS in colorectal adenocarcinoma, although they did not reach statistical significance in the GEPIA2 dataset.

These results suggest that these proteins may have prognostic value in primary gastrointestinal cancers, particularly in GC and colorectal adenocarcinoma. Further studies are needed to validate and understand the underlying mechanisms of these associations and their potential as prognostic biomarkers in gastrointestinal cancer.

In summary, this study presents a comprehensive proteomics analysis of the primary gastrointestinal cancers in KTs. Our findings provide significant proteomic landscape that these primary gastrointestinal cancers are distinct from other gastrointestinal cancers at the same anatomical site without ovarian metastases. Specifically, the dysregulation of ECM-related pathways and the aberrant expression of associated proteins highlight their critical role in tumor metastasis, offering references for identifying potential therapeutic targets. Furthermore, our study demonstrates the possibility to identify gastrointestinal cancer patients prone to developing ovarian metastases that form KT based on selected proteins. This systematic proteomic profiling characterizes the features underlying KT formation and identifies potential predictive biomarkers for these metastatic tumors, which may provide clues for early therapeutic intervention.

### Limitations of the study

Our study has some noteworthy limitations that should be considered when interpreting our results. First, the sample size of our study is relatively small due to the rarity of KT, which may affect the statistical power and generalizability of our results. The other limitation of this study is the lack of plasma samples from metastatic and non-metastatic CG and CC patients, which prevented us from exploring the diagnostic potential of the identified candidate tissue proteins in a minimally invasive plasma-based assay. Future studies should focus on collecting and analyzing plasma samples to overcome this limitation and further validate the clinical utility of these biomarkers for early detection of ovarian metastasis in gastrointestinal cancers. Additionally, we did not include other types of ovarian tumors or other sources of metastasis for comparison. Therefore, larger studies with more diverse populations are needed to validate and extend our findings. Second, although we tried our best to areas with a higher proportion of tumor components during sample collection, we did not employ methods such as laser capture microdissection to isolate pure tumor cell populations. Consequently, the presence of stromal components within the samples may have affected the results and the interpretation of the data.

## Resource availability

### Lead contact

Further information and requests for resources and reagents should be directed to and will be fulfilled by the lead contact, Bo Wang (1506128@zju.edu.cn).

### Materials availability

This study did not generate new unique reagents.

### Data and code availability

• The mass spectrometry proteomics data have been deposited to the ProteomeXchange Consortium (https://proteomecentral.proteomexchange.org/) via the iProX partner repository^62^^,^^63^ with the dataset identifier PXD072612. Also, the data are available via iProX with the following access link: https://www.iprox.cn/page/project.html?id=IPX0006036000.
• All original code has been deposited at Zenodo under https://doi.org/10.5281/zenodo.17192566 and is publicly available as of the date of publication.
• Any additional information required to reanalyze the data reported in this paper is available from the lead contact upon request.

## Acknowledgments

We thank Liming Xu, Ke Sun, Jinlong Cui, Ting Sun, and Zhihui Wang (Department of Pathology, The First Affiliated Hospital, Zhejiang University School of Medicine) for their technical support. This work is supported by grants from National Key R&D Program of China (grant no. 2022YFF0608403 to Dr. Y.Z.); Educational Project of Zhejiang Province (grant no. Y201942054 to Dr. X.W.), National Key R&D Program of China (grant no. 2021YFA1301602 to Dr. T.G.).

## Author contributions

B.W., Y.Z., and T.G., conceptualization, resources, and supervision; X.Y. and Y.Z., methodology, data curation, formal analysis, investigation, writing – original draft, and writing – review and editing; X.W., samples collection, clinical data acquisition, data analysis, and editing; W.L. and Z.X., data curation, formal analysis, methodology, and writing – review and editing; X.T., supervision and pathological diagnosis confirmation; H.Z., samples collection and clinical data acquisition.

## Declaration of interests

T.G. and Y.Z. are shareholders of Westlake Omics, Inc. W.L. is an employee of Westlake Omics, Inc.

## STAR★Methods

### Key resources table

**Table:** 

REAGENT or RESOURCE	SOURCE	IDENTIFIER
**Biological samples**		

Primary tumor with KT of gastric origin	the First Affiliated Hospital, Zhejiang University	–
Primary tumor with KT of colorectal origin	the First Affiliated Hospital, Zhejiang University	–
Gastric cancers	the First Affiliated Hospital, Zhejiang University	–
Colorectal cancers	the First Affiliated Hospital, Zhejiang University	–

**Chemicals, peptides, and recombinant proteins**		

Heptane	Sigma-Aldrich,	cat. no. 246654-2L
Formic acid (FA)	Thermo Fisher Scientific	cat. no. A117-50
Urea	Sigma-Aldrich	cat. no. U1250
Tris (2-carboxyethyl) phosphine (TCEP)	Adamas-beta	cat. no. 61820E
Iodoacetamide (IAA)	Sigma-Aldrich	cat. no. I6125
Trypsin	Hualishi Tech	cat. no. HLS TRY001C
Trifluoroacetic acid (TFA)	Thermo Fisher Scientific	cat. no. 85183
Water	Thermo Fisher Scientific	cat. no. W6-4
Acetonitrile	Thermo Fisher Scientific	cat. no. A955-4
Ethanol	Sinopharm Chemical Reagent limited corporation	cat. no. 10009218
Ammonium bicarbonate (ABB)	General-Reagent	cat. no. G12990A
Lys-C	Hualishi	cat. no. HLS LYS001C
HCl	Hualishi	cat. no. HLS HCL001C
Acetonitrile(ACN)	Fisher Chemical	cat. no. A955-4

**Deposited data**		

The proteomics data	This paper	https://proteomecentral.proteomexchange.org/ via the iProX partner repository with the dataset identifier PXD072612. https://www.iprox.cn/page/project.html?id=IPX0006036000

**Software and algorithms**		

Proteome Discoverer v2.5.0.400	–	Thermo Fisher Scientific
pFind v3.1.5	–	Institute of Computing Technology, Chinese Academy of Sciences
DIA-NN (Version 1.6.0)	Demichev et al.^20^	https://github.com/vdemichev/diann
All original code	This paper	https://doi.org/10.5281/zenodo.17192566
SPSS version 13.0	SPSS Inc., Chicago, IL, USA	–
Cytoscape(Version 3.8.2)	Institute for Systems Biology	–

**Other**		

The GEPIA2 database	Su et al.	http://gepia2.cancer-pku.cn
DIA mode on the DIONEX UltiMate 3000 nano System	Thermo Fisher Scientific™, San Jose, USA	–
Q Exactive HF hybrid Quadrupole-Orbitrap	Thermo Fisher Scientific™, San Jose, USA	–

### Experimental model and study participant details

The formalin-fixed paraffin-embedded (FFPE) tissue samples in this study were acquired from two specific cohorts: the training cohort and the test cohort.

In the training cohort, we collected formalin-fixed paraffin-embedded (FFPE) tumor tissue samples from 23 patients diagnosed with gastric-derived KT tumors and 22 patients diagnosed with colorectal-derived KT tumors between January 2007 and June 2017. Additionally, we included 45 cases of primary gastric cancer and 50 cases of primary colorectal cancer during the same period. A total of 185 FFPE tumor tissue samples were collected, including 45 biological replicates.

For the test cohort, samples were procured from 9 patients with gastric-origin KT and 17 patients with colorectal-origin KT, which included primary tumors and the corresponding ovarian tissues, from May 2013 to June 2020. To enable comparison, we also collected data from 16 primary gastric cancer cases and 36 primary colorectal adenocarcinoma cases, none of which had ovarian metastasis, during this period. The inclusion criteria for control cases paralleled those set for the training cohort. All the patients are female, of Han ethnicity and Chinese nationality.

The Ethics Committee of the First Affiliated Hospital, Zhejiang University School of Medicine, approved this study, under approval number 2017-600-1(Data S1). The data analysis was conducted anonymously, so the Committee waived the written informed consent requirement. All experiments were carried out in strict compliance with relevant guidelines and regulations.

The sampling protocol for FFPE tissue specimens is as follows: Initially, the proportion of tumor cells is assessed via microscopic examination of Hematoxylin and Eosin (HE)-stained sections. Regions exhibiting a high density of tumor cells with minimal stromal content (as illustrated in Figure S1) are selected and marked. Subsequently, corresponding areas on the paraffin blocks are re-marked. Utilizing a multi-point sampling approach, at least 2 representative regions per specimen are sampled using tissue microarray techniques, and the resulting tissue cores are transferred into sterile Eppendorf (EP) tubes.^64^

### Method details

#### Protein extraction and peptide preparation

The tissue samples were processed for proteomic analysis as previously described using the PCT-assisted method.^65^^,^^66^^,^^67^ Briefly, the FFPE samples were first de-paraffinized by heptane, re-hydrated by successive ethanol series (100% ethanol, 90% ethanol, and 75% ethanol), and hydrolyzed by 0.1% FA and 0.1 M Tris-HCl (pH = 10.0). Then the samples were processed with pressure cycling technology (PCT)-assisted lysis and digestion. The sample was lysed in a lysis buffer (6M urea, 2M thiourea 0.1M ammonium bicarbonate). The extracted proteins were then reduced and alkylated by tris(2-carboxyethyl) phosphine (final concentration 10 mM) and iodoacetamide (final concentration 40 mM) respectively. Then the proteins were digested by Lys-C at an enzyme-to-substrate ratio of 1:40 (wt/wt) and Trypsin at an enzyme-to-substrate ratio of 1:50 (wt/wt). Peptide samples were acidified with trifluoroacetic acid (TFA) to terminate enzyme digestion, then desalted with C18 and stored at −80°C until mass spectrometry analysis.

#### DIA-MS analysis

Peptide analyses were performed in DIA mode on the DIONEX UltiMate 3000 nano System coupled with the Q Exactive HF hybrid Quadrupole-Orbitrap (Thermo Fisher Scientific, San Jose, USA) as described previously.^65^ Briefly, all peptide samples were separated in 60 min (75 min total from injection to injection) at a flow rate of 300 nL/min over a linear gradient of 3–28% (buffer A: 2% ACN, 0.1% FA, buffer B: 98% ACN, 0.1% FA). A full MS scan was obtained using an AGC target value of 3E6 charges and a maximum IT of 80 ms in the orbitrap, analyzing 390–1010 m/z with a resolution of 60,000 (at m/z 200). After the MS scan, 24 MS/MS scans were obtained, a resolution of 30,000 at m/z 200, AGC target 1E6 charges, normalized collision energy of 27%, default charge state set to 2, and maximum IT set to auto.

#### Proteomic data processing and analysis

The DIA-MS data search was conducted using DIA-NN (Version 1.6.0)^20^ following the official manual against the DPHL spectral library (comprising 10,943 proteins, accessed on August 12, 2020).^68^ The precursor false discovery rate was set as 0.01.

#### Gene set enrichment analysis (GSEA) analysis

To acquire comprehensive information on the signaling pathway changes at the protein level, we performed GSEA analysis, using the java GSEA Desktop Application (v4.1.0). The relative protein abundance measured from KTGC/GC and KTCC/CC groups were input as expression datasets. The enrichment pathways were matched against “c2.cp.v7.2. symbols” gene set database. Normalized enrichment score (NES) and nominal *p*-value estimation were the primary statistical methods to examine the genomic enrichment results. The GESA-enriched pathways were visualized by Cytoscape software (v3.8.2).

#### Differential expression analysis

For more rigorous analysis, in the KTGC/GC and KTCC/CC groups, the proteins that are not detectable in more than 70% of the samples are removed. The missing values of the protein matrix were imputed as zero. Protein fold change (FC) between the two groups was calculated using the relative protein abundance. The *p*-value was calculated by a two-sided unpaired *t* test using log2 relative protein abundance between groups. All the *p*-value were performed Benjamini & Hochberg correction (adjusted *p*-value). Proteins with adjusted *p* value less than 0.05 and absolute log_2_ (FC) over 0.5 were regarded as significantly changed proteins.

#### Machine learning

R package, RandomForest (version 4.6.14), was used to build a classification model that distinguishes between KTGC and GC, or between KTCC and CC. For the model building, the ntree was set as 1000, and the node size was 1. The AUC and diagnostic accuracy were evaluated using the R package pROC (version 1.18.5) to assess the performance metrics of the model.

#### GEPIA2 database

The GEPIA2 database^55^ (http://gepia2.cancer-pku.cn/) was utilized to assess the expression and prognosis of the model genes in patients with gastric cancer and colorectal adenocarcinoma. The screening modules and main analysis conditions were as follows: the survival analysis module was employed for expression analysis, with “Gene”, “Overall Survival”, “Median”, “Yes” selected for Hazards Ratio (HR), and “Yes” for the 95% Confidence Interval in Survival Plots. In addition, “STAD” was chosen as the dataset for gastric cancer, while “COAD” and “READ” were selected as the datasets for colorectal adenocarcinoma.

### Quantification and statistical analysis

The clinicopathological characteristics of primary gastric and colorectal cancers with and without ovarian metastasis in both the training and test cohorts were compared using the chi-square test. The statistical analyses were performed using SPSS version 13.0 (SPSS Inc., Chicago, IL, USA), and a two-tailed *p* value <0.05 was considered statistically significant. Statistical analysis of DIA mass spectrometry data was performed with R (version 4.4.1).

### Additional resources

Description: No additional resources are available beyond those included in the article.

## Supplemental information

Document S1. Figures S1–S5 and Tables S1 and S2

Data S1. Approval letter of Research Ethics Committee of the First Affiliated Hospital, College of Medicine, Zhejiang University

Table S3. Patients and sample information

Table S4. The protein matrix and peptide matrix for all samples

Table S5. Differentially expressed proteins
