# Supplementary material for: Proteomic profiling identifies prognostic signature for Krukenberg tumor of gastrointestinal origin
Source: iScience. 2026 Jan 13;29(2):114682. doi: 10.1016/j.isci.2026.114682 (PMC12907060; doi:10.1016/j.isci.2026.114682)
Supplement: Document S1. Figures S1–S5 and Tables S1 and S2 [file mmc1.pdf]

iScience, Volume *29*

Supplemental information

Proteomic profiling identifies prognostic signature for Krukenberg tumor of gastrointestinal origin

Xiaoling Wang, Xiao Yi, Zhangzhi Xue, Wei Liu, Xiaodong Teng, Han Zhang, Tiannan Guo, Yi Zhu, and Bo Wang

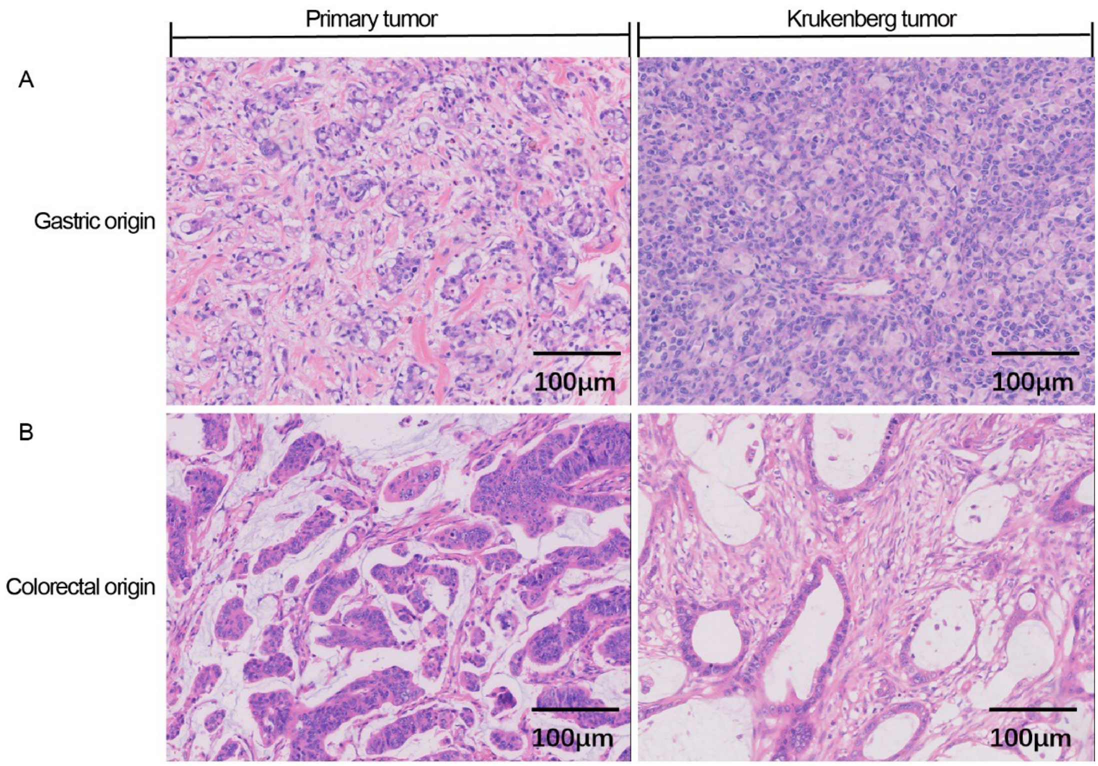

Figure S1. Representative hematoxylin and eosin (H&E) images of Krukenberg tumors and corresponding primary cancers. A.) A case of Krukenberg tumor from gastric cancer, including primary gastric tumor (left) and ovarian metastasis (right). B.) A case of Krukenberg tumor from colorectal cancer, including primary colorectal tumors (left) and ovarian metastasis (right).

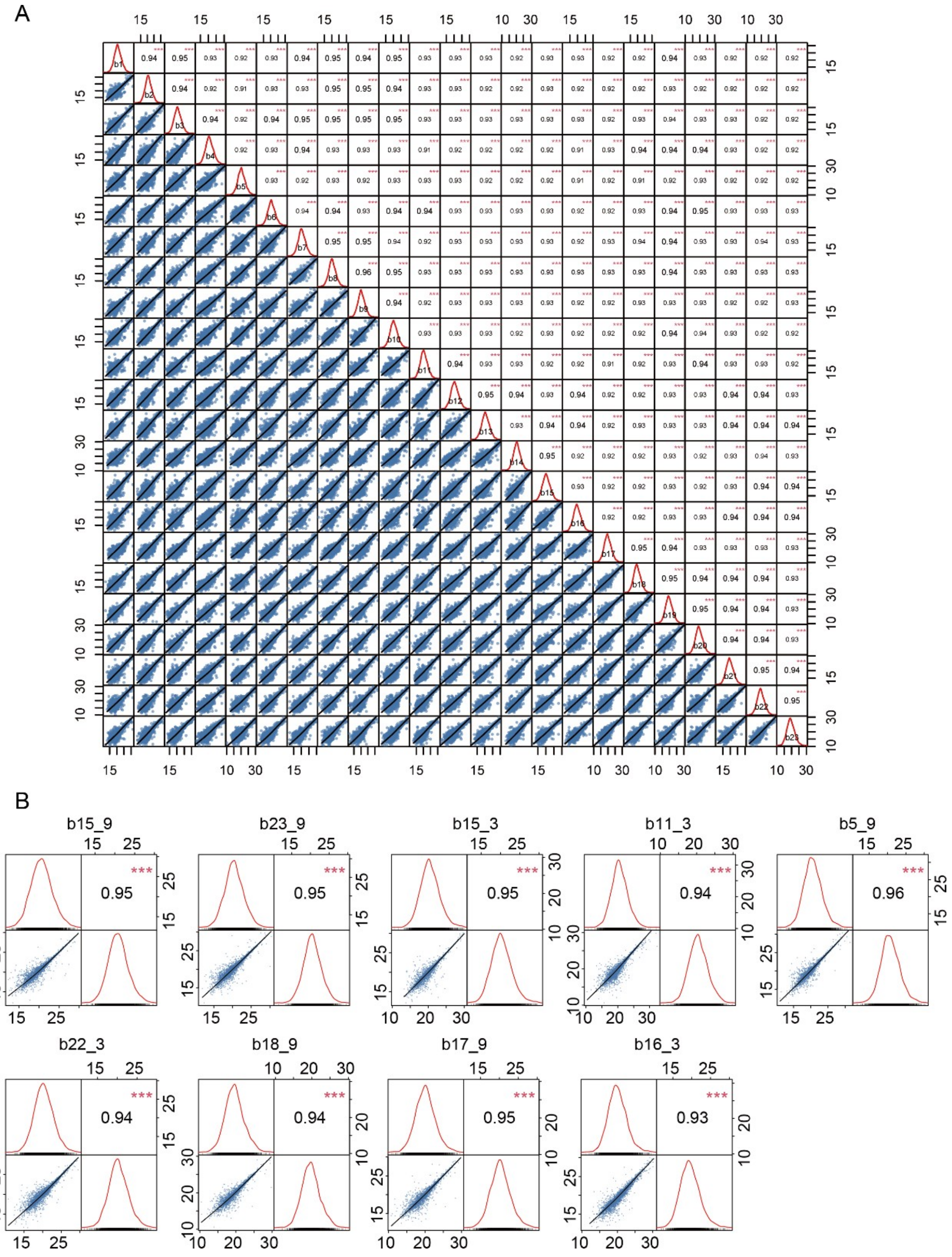

Figure S2. Quality control of proteomic data. A.) Pearson correlation analysis of 23 pool samples. B.) Pearson correlation analysis of 9 technical replicated pairs. The top-left and bottom-right panels represent the density of log2-transformed protein intensity in two technical replicates, while the bottom-left panel shows the linear fit of protein intensity within the technical replicate. The numerical value in the top-right panel displays the Pearson correlation coefficient for protein intensity in two technical replicates. The asterisk in the top-right panel indicates the p-value based on Pearson's product-moment correlation coefficient. p-value: $*p < 0.05$; $**p < 0.01$; $***p < 0.001$.

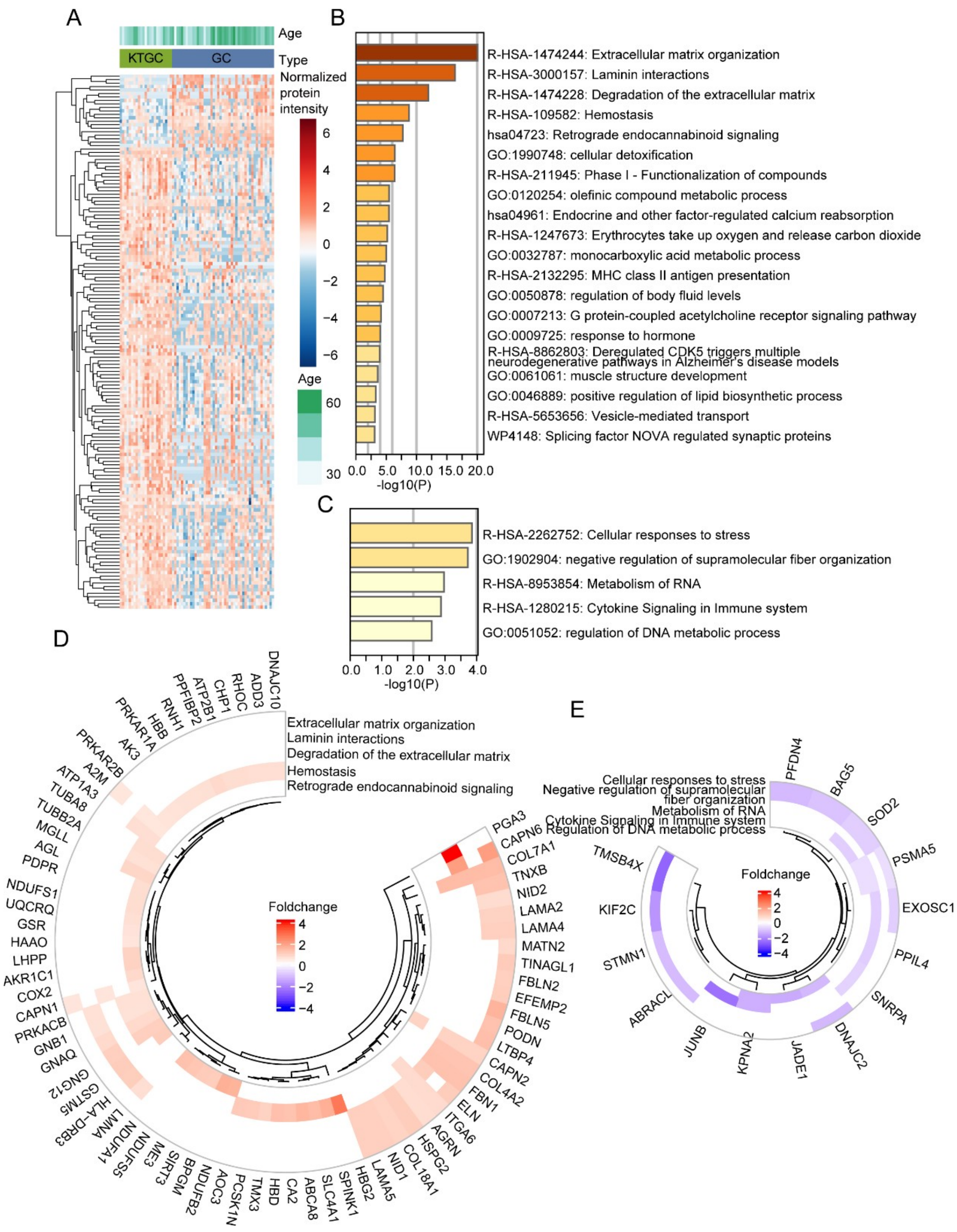

Figure S3. Dysregulated protein expression analysis in KTGC and GC. A.) Heatmap overview of significantly regulated proteins in KTGC versus GC. Each row means a protein and each column means a patient. Proteins abundance is Z-score normalized. Pathway enrichment of the up-regulated (B) and down-regulated (C) proteins in KTGC. Bar graph of enriched terms across input protein list, colored by p-values. The circular heatmap shows that up-regulated (D) and down-regulated (E) proteins are involved in the top five enriched pathways in KTGC.

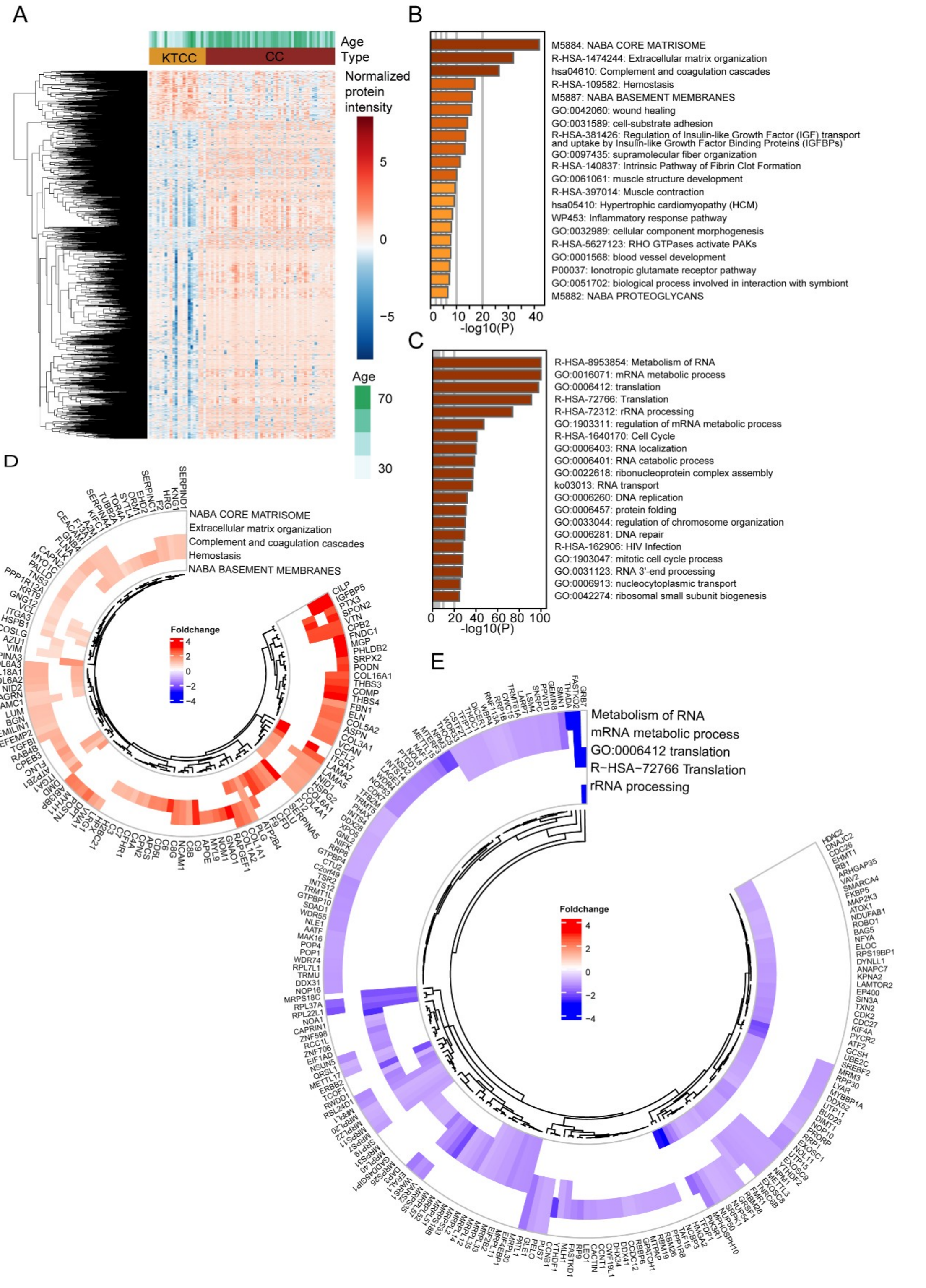

Figure S4. Dysregulated protein expression analysis in KTCC and CC. A.) Heatmap overview of significantly regulated proteins in KTCC versus CC. Pathway enrichment of the up-regulated (B) and down-regulated (C) proteins in KTCC. Bar graph of enriched terms across input protein list, colored by p-values. The circular heatmap shows that up-regulated (D) and down-regulated (E) proteins are involved in the top five enriched pathways in KTCC.

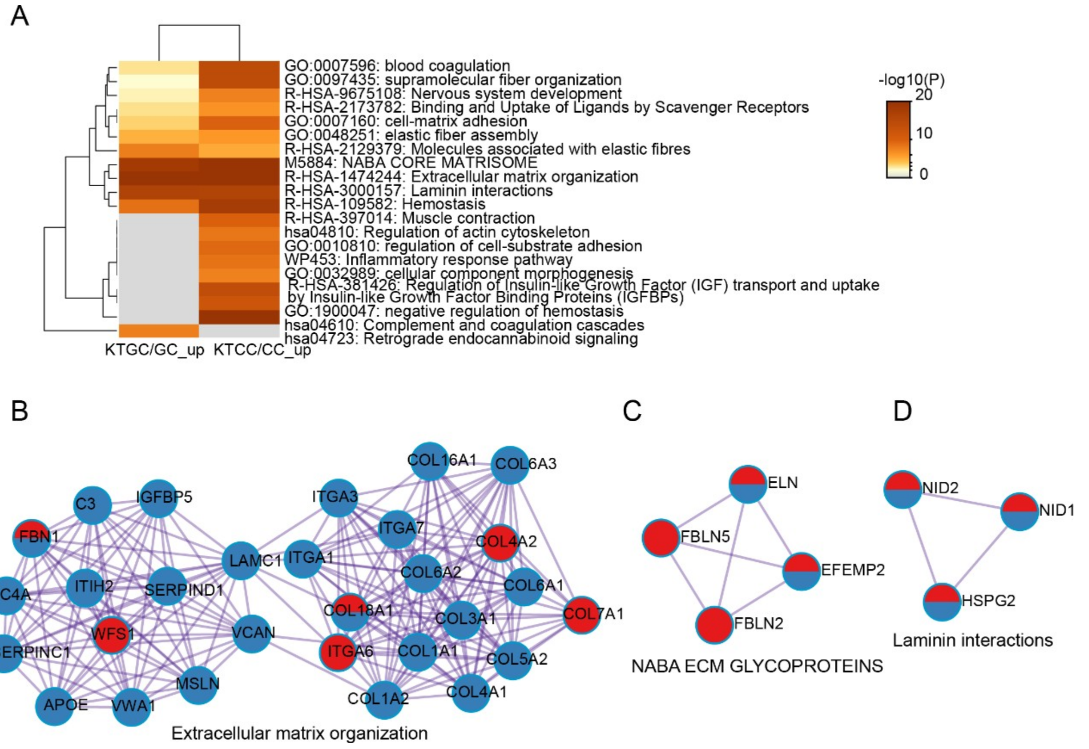

Figure S5. Overlap analysis across KTGC/GC and KTCC/CC. A.) Heatmap shows the top 20 enrichment pathways, using a discrete color scale to represent statistical significance. The gray color indicates a lack of significance. B-D.) The network shows that processes such as Extracellular matrix organization (B), ECM glycoproteins (C), and Laminin interactions (D) are generally shared among the two lists. Red represents this protein upregulated in KTGC. Blue represents this protein upregulated in KTCC.

Table S1 Clinicopathological features of primary gastric cancers with and without ovarian metastasis in training and test cohort

	Training cohort			Test cohort		
	KTGC	GC	P	KTGC	GC	P
Age (y)						
≥60	0 (0)	5(11.1%)	0.159*	0 (0)	7 (43.8%)	0.027*
< 60	23 (100%)	40(88.9%)		9 (100%)	9 (56.2%)	
Menopause						
Pre	20 (87.0%)	21(46.7%)	0.001	6 (66.7%)	7 (43.8%)	0.411*
Post	3 (13.0%)	24 (53.3%)		3 (33.3%)	9 (56.2%)	
Chronology						
Metachronous	11 (47.8%)	NA		8 (88.9%)	NA	
Synchronous	12 (52.2%)	NA		1 (11.1%)	NA	
Bilaterality						

Bilateral	15 (65.2%)	NA		8 (88.9%)	NA	
Unilateral	8 (34.8%)	NA		1 (11.1%)	NA	
Signet-ring cells						
Present	17 (73.9%)	17(37.8%)	0.005	3 (33.3%)	10 (62.5%)	0.226*
Absent	6 (26.1%)	28(62.2%)		6 (66.7%)	6 (37.5%)	
Differentiation						
Poor	23 (100.0%)	36(80.0%)	0.023*	9 (100.0%)	15 (93.8%)	1.000*
Well/Moderate	0 (0)	9(20.0%)		0 (0)	1 (6.2%)	
Lymph node status						
Positive	21 (91.3%)	33(73.3%)	0.116*	9 (100.0%)	15 (93.8%)	1.000*
Negative	2 (8.7%)	12(26.7%)		0 (0)	1 (6.2%)	

*: Fisher's exact test. NA: not applicable.

Table S2 Clinicopathological features of primary colorectal cancers with and without ovarian metastasis in training and test cohort.

	Training cohort			Test cohort		
	KTCC	CC	P	KTCC	CC	P
Age(y)						
≥60	5 (22.7%)	19 (38.0%)	0.205	7 (41.2%)	15 (41.7%)	0.973
< 60	17 (77.3%)	31 (62.0%)		10 (58.8%)	21 (58.3%)	
Menopause						
Pre	15(68.2%)	14 (28.0%)	0.001	7 (41.2%)	15 (41.7%)	0.973
Post	7(31.8%)	36 (72.0%)		10 (58.8%)	21 (58.3%)	
Chronology						
Metachronous	10 (45.5%)	NA		9 (52.9%)	NA	
Synchronous	12 (54.5%)	NA		8 (47.1%)	NA	
Bilaterality						

Bilateral	14 (63.6%)	NA		5 (29.4%)	NA	
Unilateral	8 (36.4%)	NA		12 (70.6%)	NA	
Signet-ring cells						
Present	2 (9.1%)	0 (0)	0.090*	1 (5.9%)	1 (2.8%)	0.543*
Absent	20 (90.9%)	50 (100%)		16 (94.1%)	35 (97.2%)	
Differentiation						
Poor	13 (59.1%)	15(30.0%)	0.020	4 (23.5%)	16(44.4%)	0.143
Well/Moderate	9 (40.9%)	35(70.0%)		13 (76.5%)	20(55.6%)	
Lymph node status						
Positive	17 (77.3%)	21 (42.0%)	0.006	14 (82.4%)	16(44.4%)	0.009
Negative	5 (22.7%)	29 (58.0%)		3 (17.6%)	20(55.6%)	

*: Fisher's exact test. NA: not applicable.
