# Supplementary material for: Proteomic profiling identifies prognostic signature for Krukenberg tumor of gastrointestinal origin
Source: iScience. 2026 Jan 13;29(2):114682. doi: 10.1016/j.isci.2026.114682 (PMC12907060; doi:10.1016/j.isci.2026.114682)
Supplement: Data S1. Approval letter of Research Ethics Committee of the First Affiliated Hospital, College of Medicine, Zhejiang University [file mmc2.pdf]

加快审查类

浙江大学医学院附属第一医院 科研伦理审查委员会 伦理审查批准件
Approval Letter of Research Ethics Committee of the First Affiliated Hospital, College of Medicine, Zhejiang University

批件号 Reference Number：（ 2017）科研快审第（ 600-1 ）号

研究方案名称 Study Title	关于 Krukenberg 瘤原发灶、淋巴结转移灶和卵巢转移灶之间蛋白、基因表达差异的研究		
承担学科 Department	病理学	项目负责人 P.I.	王波
审批材料清单 List of Documents	■审查申请表 Application form<br>■研究方案 Protocol<br>☐知情同意书 Informed consent form<br>☐其它 Other________________	*组长单位（注：非本院牵头项目需填）<br>Lead Site	

审查意见 Evaluation Comments：

■批准 Approval

☐作必要修改后批准 Conditional Approval

☐不批准本次申请 Disapproval

伦理委员会主任或副主任签名：
Signature of Chairman of the Ethics Committee 郑敏

日期： 2017 年 08 月 02 日
Date

浙江大学医学院附属第一医院
科研伦理委员会
Research Ethics Committee of the First Affiliated Hospital,
College of Medicine, Zhejiang University

注：本审查意见仅对本次项目审批有效，如内容有变动需再次审批。

Annotate: This review comments is only effective for the project examination and approval. If the project has any change, it must be examined and approved again.

备注:

1. 研究者应遵循伦理委员会批准的方案执行，实施过程应符合赫尔辛基宣言的原则。
2. 在试验实施过程中，对研究方案和知情同意等相关文件所作的任何修改，均需得到伦理委员会审查同意后方可实施。
3. 发生严重不良事件及可能影响风险受益比的任何事件和新信息须及时报告本院伦理委员会。
4. 接受伦理委员会持续审查的项目，请在到期前1个月（无论试验开始与否）提出再次审查的申请。
5. 如有不依从/违背方案或暂停/提前终止的试验项目，应及时以书面文件报告本院伦理委员会；临床试验结束后，须及时向伦理委员会提交结题报告。
6. 本批件有效期 1 年（自批准之日起），如试验逾期未实施即自行废止。
